# An Historical View of Surgery in the Sixteenth Century

**Published:** 1799-10

**Authors:** 


### *An Historical View of Surgery in the Sixteenth Century.*

[Continued from our last Number, pp. 155—160.

§ 12. TO become acquainted with the most celebrated surgeons of that century, we shall proceed in chronological order. One of the oldest chirurgical writers is HIERON BRAUNSCHWEIG, a surgeon who practised at Strasburg. His book contains but few original principles, as he does not enter upon the theory, and points out the means as well as the manual operations, rather in a mechanical manner. On the treatment of ulcers his ideas are generally correct; he does not absterge the pus with much solicitude, but, on the contrary, considers it as a healing balsam. He mentions a case of hydrophobia, the symptoms of which became manifest after twelve months had elapsed since the bite of the dog; and against which he prescribed cantharides internally. The external remedies he usually applied, were regulated conformably to the difference of climate, so that in a moist climate he used absorbents, while in a warm climate, he directed humid applications. In depressions of the cranium, he recommends an ointment of the white of eggs and oxycroceum, which he believed to be very efficacious.

The name of JOHN DE VIGO, a native of Rappali, in Genoa, and physician to the Pope, is no less celebrated. He wrote two Compendiums of Surgery, and I have already observed, that he was no advocate for operations; but he is the more liberal in his praise of medicinal substances, for instance, of a solution of white vitriol in rose-water, in epiphora; of the oleum elemi, &c. in wounds of the nerves. His literary knowledge was extremely defective, and his method of treating external diseases much too rash, as he was too profuse in the administration of wine. Yet we meet occasionally with interesting remarks in his works. He opens abscesses by the semilunar incision, gives a pretty correct account of the causes of gangrene, and teaches us to treat it with the actual cautery. He extirpated an encysted tumour under which the Pope had laboured, by the Egyptian ointment and sublimate. In a similar manner he treated scrophulous tumours, and the whitlow, while he likewise applied the actual cautery for these diseases, as well as for the fistula lachrymalis. According to the old practice, he began amputation with making an incision into the mortified part; but at the same time he dissuades the practitioner from prescribing opium during the operation. The doctrine relative to concussions of the brain, he has delivered with tolerable accuracy for those times; and he also observed, that bleeding of the nose in this case was critical. He attempted to cure wounds of the head merely with absorbent remedies; but he does not fail likewise likewife to recommend the application of the trepan as fpeedily as poffible. On account of the double membranes of the brain, he objects to placing the trepan on the futures of the cranium, as he had frequently obferved, that, after wounds of the head were apparently healed, they were attended with a concealed inflammation of the dura and pia-mater, or of the cortical fubftance of the brain.

§ 13. The treatment of wounds would have experienced an entirely new epocha, if MICHAEL ANGELO BIONDO, of Venice, who practifed fucceffively at Naples, Venice, and Rome, had been a man of fufficient celebrity. He firft recommended *cold water* to be indifcriminately ufed as the beft remedy in all kinds of wounds, excepting thofe of the nerves and contufions; while he expected miraculous effects from this remedy, which it certainly has produced, according to modern experience, in numberlefs cafes of injuries of the head. Indeed, Biondo attributed to his *oleum abietinum* almoft equally powerful effects; but his book was too defective in compofition and arrangement to be entitled to general approbation.

The large chirurgical work of JOH. ANDR. DA CROCE, had no ftronger claim to profeffional fupport than the former. The author, who practiced at Venice, was a mere compiler from the medical works of the Arabians: he alfo recommended the trepan in all cafes of fractures of the cranium.

The doctrine refpecting the injuries of the head, is however much indebted for its improvements to JAC. BERENGAR DE CARPI *, with whofe character, as a diftinguifhed anatomift, the reader will become acquainted in the fequel. He was the firft who expofed the fallacy of the ufual fymptoms formerly obferved in fractures of the cranium; for feveral cafes had occurred to him in which the patients could bear confiderable concuffions. He doubted the reality of reciprocal or oppofite fractures, when the power operates only on one fide; but he obferved a fracture of the interior table of the cranium, though the exterior table had been uninjured. He believed that depreffions of the cranium could be healed by plafters; and afcribed moft of the unfavourable fymptoms arifing from injuries of the head to the fplinters of bones which irritated the brain and its membranes. In all fuch cafes, he above all things, recommends the oil of rofes, as well as the oil obtained from the hufks of the grape, and likewife the dyers' weed.

§ 14. MARIANO SANTO DE BARLETTA, whom we already know as a celebrated lithotomift, practifed furgery at Naples, and wrote, among other works,

* *Berengar, de Fracturis Cranii.* 8vo. Lugd. Batav. 1651.

works, a commentary on fome of the chirurgical works of EBN SINA. In thefe we find indeed much aftrological jargon, and an uncommon animofity againft phyficians who interfere with the practice of furgery, without underftanding the application of ointments, and the ufe of mercury: but Mariano Santo has neverthelefs acquired great applaufe for banifhing feveral prejudices which prevailed in the treatment of wounds, and which had even been fanctioned by the names of eminent writers. He oppofed the ufe of all cold and aftringent remedies in bruifes and eryfipelas. He particularly attacks Berengar, on the mifapplication of the oil of rofes in wounds of the head, and recommends in place of it, fpirit of wine. He alfo ridicules the idea of filling up the depreffions of the cranium and preventing dangerous confequences, by plafters: on the contrary, he affirms that the patient will die apoplectic, before this remedy can have any effect. In fractures of the cranium, he entirely rejects the chiffel and the hammer, and fupports his cenfure with very conclufive arguments. Laftly, he does not attempt to ftop the hemorrhage by applying the actual cautery like his predeceffors, but by a proper ligature.

The great anatomift GABRIEL FALLOPIUS, of whom we fhall farther treat in the fequel, was likewife an experienced furgeon. Although he adhered too clofely to the opinions of his predeceffors, yet his works contain many important principles relative to the treatment of chirurgical cafes. He was indeed no friend of the chiffel for removing the fractured bones of the cranium, and likewife advifed the fpeedy application of the trepan, even previous to the fourth day, yet he not only recommended the cold and aftringent applications too generally, but likewife formed too fanguine expectations from internal remedies. He occafionally removed large pieces from the cortical fubftance of the brain, without experiencing any ill effects. In humid ulcers he ufed alum-water, and in the caries offium, he applied the actual cautery. The amputation itfelf he performed with red-hot inftruments, and advifes to burn the diffected veffels afterwards feparately. In other cafes of hemorrhages, however, he difapproves of the expedient of burning, and gives the preference to the ligature. Againft gangrene he ufes arfenic and fublimate; in luxations he condemns the ufe of cerates, and recommends fimple humectation of the bandage with cold water. He propofes the melliferous moifture which fettles on the leaves of the elm-tree, as an excellent vulnerary, and highly praifes the virtues of pure olive oil, in the cure of wounded nerves. In the fiftula lachrymalis he does not perforate the offa lachrymalia, but performs the operation with a fyringotom, and endeavours to remove the callus with Egyptian ointment. In very protruding ruptures, he cauterizes the annulus abdominis, to produce an efchar, eschar, thus to slough off the ring, and to enable him the more effectually to reduce the protruded parts. In cancer he makes use of arsenic and oil of roses; he also extirpates the cancerous ulcer, and destroys its roots by the actual cautery.

[To be continued in our next Number.]

